# Genome-wide joint analysis of single-nucleotide variant sets and gene expression for hypertension and related phenotypes

**DOI:** 10.1186/s12919-016-0017-x

**Published:** 2016-10-18

**Authors:** Xiaoran Tong, Changshuai Wei, Qing Lu

**Affiliations:** 1 Department of Epidemiology and Biostatistics, Michigan State University, East Lansing, MI 48824 USA; 2 Health Science Centre, University of North Texas, Fort Worth, TX 76107 USA

## Abstract

**Background:**

With the advance of next-generation sequencing technologies, the study of rare variants in targeted genome regions or even the whole genome becomes feasible. Nevertheless, the massive amount of sequencing data brings great computational and statistical challenges for association analyses. Aside from sequencing variants, other high-throughput omic data (eg, gene expression data) also become available, and can be incorporated into association analysis for better modeling and power improvement. This motivates the need of developing computationally efficient and powerful approaches to model the joint associations of multilevel omic data with complex human diseases.

**Methods:**

A similarity-based weighted U approach is used to model the joint effect of sequencing variants and gene expression. Using a Mexican American sample provided by Genetic Analysis Workshop 19 (GAW19), we performed a whole-genome joint association analysis of sequencing variants and gene expression with systolic (SBP) and diastolic blood pressure (DBP) and hypertension (HTN) phenotypes.

**Results:**

The whole-genome joint association analysis was completed in 80 min on a high-performance personal computer with an i7 4700 CPU and 8 GB memory. Although no gene reached statistical significance after adjusting for multiple testing, some top-ranked genes attained a high significance level and may have biological plausibility to hypertension-related phenotypes.

**Conclusions:**

The weighted U approach is computationally efficient for high-dimensional data analysis, and is capable of integrating multiple levels of omic data into association analysis. Through a real data application, we demonstrate the potential benefit of using the new approach for joint association analysis of sequencing variants and gene expression.

## Background

Next-generation sequencing technology provides denser genetic profiles than previous microarray-based genotyping technology [1]. It could effectively capture rare variants with low minor allele frequency (MAF). Driven by the advance of sequencing technology and limited heritability explained by the genome-wide association studies (GWAS) findings [2, 3], current research focus has shifted toward studying rare variants associated with common complex diseases. Although these studies hold great promise for finding new genetic variants predisposing to human disease, they also face great challenges, for example, low power for detecting rare variants because of their low frequency. The dramatic increase in numbers of single nucleotide variants (SNVs) also raises computational and statistical challenges (eg, multiple testing issue). One practical strategy is to group multiple SNVs according to known functional information (eg, variants in a gene or a pathway) or location (eg, variants in a fix-sized bin [4]), and jointly analyze these SNVs [5, 6]. By grouping and testing multiple SNVs, we are able to aggregate association signals and reduce the number of tests.

Besides SNVs, other omic data, such as gene expression, could also be collected. These intermediate omic data can be integrated into sequencing studies for improved power and better biological interpretation. While the conventional analysis only links SNVs or gene expression to disease phenotypes, the emergence of multilevel data brings the possibility of jointly analyzing SNVs and other omic data. By fully utilizing the information, the joint analysis has great potential to improve power [7]. Nevertheless, how to efficiently analyze the high-dimensional sequencing data and other omic data remains a challenge.

## Methods

In this empirical study, we used a similarity based weighted U approach to jointly model SNVs and gene expression data of 142 unrelated Mexican American samples provided by Genetic Analysis Workshop 19 (GAW19). By using the weighted U approach, we performed a genome-wide joint association analysis, evaluating the association of 17,558 genes with three phenotypes (ie,, systolic blood pressure [SBP], diastolic blood pressure [DBP], and hypertension [HTN]).

For the integrative analysis, we extended previously developed nonparametric approaches [8] to handle both SNVs and gene expression. To aggregate the rare variants in a gene, a weighted sum approach is used [8]. Let *p*_*k*_ denote the MAF of the *k* ^th^ SNV (*k* = 1,2,…,*K*), the weight for the *k* ^th^ SNV can be defined as ${w}_k=1/\sqrt{p_k\left(1-{p}_k\right)}$. Let *K* be the total number of SNVs in a gene region, the weighted sum score for the *j* ^th^ sample can be obtained by,

$$ {a}_j=\frac{{\displaystyle {\sum}_{k=1}^K{w}_k{v}_{jk}}}{2{\displaystyle {\sum}_{k=1}^K{w}_k}}, $$

where *v*_*jk*_ is the genotype value of the *k* ^th^ SNV for the *j* ^th^ sample, coded by the minor allele count (ie, 0, 1, and 2). We then define a weighted U statistic to assess the joint effect of SNVs and gene expression on the disease phenotype,

$$ U={\sum}_{i\ne j}f\left({a}_i,{a}_j\right)\mathit{\mathsf{g}}\left({t}_i,{t}_j\right)h\left({y}_i,{y}_j\right), $$

where *f*(*a*_*i*_,*a*_*j*_), *g*(*t*_*i*_,*t*_*j*_), and *h*(*y*_*i*_,*y*_*j*_) measure the similarities of SNVs, gene expression, and phenotypes, respectively. Phenotypic similarity *h*(*y*_*i*_,*y*_*j*_) serves as the U kernel,

$$ h\left({y}_i,{y}_j\right)=\frac{\left({y}_i-E(Y)\right)\left({y}_j-E(Y)\right)}{Var(Y)}, $$

where *y*_*i*_ and *y*_*j*_ are ranks of the *i* ^th^ and *j* ^th^ samples’ phenotypes. The genetic and gene expression similarities are weight functions, defined based on the Gaussian distance,

$$ f\left({a}_i,{a}_j\right)={e}^{-\frac{{\left({a}_i-{a}_j\right)}^2}{2N}}\kern0.5em \mathit{\mathsf{g}}\left({t}_i,{t}_j\right)={e}^{-\frac{{\left({t}_i-{t}_j\right)}^2}{2N}}, $$

where *a*_*i*_ (*a*_*j*_) and *t*_*i*_ (*t*_*j*_) denote the weighted sum score and the gene expression value of the individual *i*(*j*), respectively.

Under the null hypothesis of no association, phenotypic similarity is unrelated to genetic or gene expression similarities. Because phenotypic similarity is symmetric, that is, *E* (*h*(*y*_*i*_,*y*_*j*_)) = 0, the expectation of U statistic is 0. Under the alternative, phenotypic similarity increases with the increase of genetic or gene expression similarities. Therefore, the positive phenotypic similarities are heavier weighted and the negative phenotypic similarities are lighter weighted, leading to a positive value of U. Because the U kernel satisfies the finite second moment condition, *E*(*h* ^*2*^(*y*_*i*_ *,y*_*j*_)) < ∞, and is degenerate (ie, *Var*(*E*(*h*(*y*_*i*_,*y*_*j*_))) = 0), the limiting distribution of U can be approximated as a linear combination of chi-squared random variables with one degree of freedom [8], and its *p* value can be obtained by using the Davis method [9].

The weighted U approach is also flexible for testing other hypothesis. In addition to evaluating the joint effect of genetic markers and gene expression (G + T), it could be used for testing genetic effect (G) alone or gene expression (T) effect alone. For example, we can modify the approach by setting the gene expression similarity as constant (eg, *g*(*t*_*i*_,*t*_*j*_) ≡ 1) to test genetic effect.

## Results

### Genome screening

We applied three tests (ie, G + T, G, and T) to 142 unrelated Mexican American samples from the San Antonio Family Heart Study (SAFHS) and the San Antonio Family Diabetes/Gallbladder Study (SAFDGS). All analyses were based on SNVs on the odd-numbered autosomes and gene expression data provided by GAW19. In this study, we assembled multiple SNVs based on the functional unit (ie, gene) to facilitate the joint modeling of gene and gene expression. We obtained primary and alternative assembles from Genome Reference Consortium release version 38 (GRCh38) and identified 32,436 gene regions in correspondence to 17,264 RNA probes. The number of gene regions exceeds the probes because multiple assembles of one gene can share one nucleotide sequence, as well as the RNA probes designed to capture such sequence. SNVs that are not within or near a gene (±5 kb at both ends) were removed. Gene regions with no SNVs or RNA probes were also discarded. SNVs with no variation (ie, MAF = 0) were dropped, as were gene regions containing only such SNVs. A total of 6,956,910 SNVs, corresponding to 17,558 gene regions, remained for the joint analysis. The first, second, and third quartiles of the SNV counts in these regions are 115, 205, and 411, respectively. We used SBP, DBP, and HTN measurements at the first examination year as phenotypes, and age, gender, medication use, and smoking status as covariates. To account for population stratification, we performed principal components (PCs) analysis by using the EIGENSTRAT software [10]. The first 20 PCs were used in the analysis to adjust for potential confounding bias because of population stratification.

**Fig. 1:**
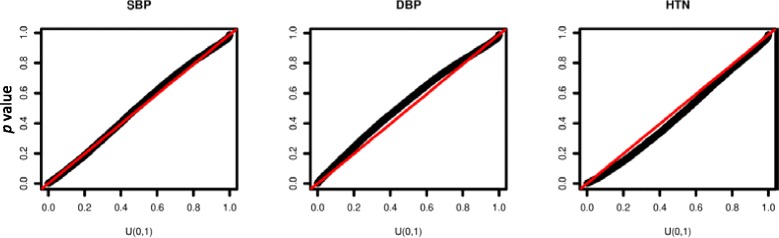
QQ plot for the joint association analysis (G + T) of SBP, DBP, and HTN

The whole-genome joint analysis of 3 phenotypes was completed in 80 min using a single core of i7 4700 CPU with 8 GB memory. Table 1 summarizes the top genes from the analysis, which were selected based on the smallest *p* value of three tests. In general, we observed that the G + T test either attained the smallest *p* value or a *p* value close to the smallest one. After adjusting for multiple testing, none of the genes were significantly associated with the phenotypes. However, if we used a significant threshold of 0.05, 4 of 15 genes were missed by considering SNVs alone (ie, G) and six genes were missed by considering gene expression alone (ie, T), while all 15 genes could be captured by the joint association analysis (ie, G + T). This suggests that there are potential advantages to combining genetic and gene expression information in the association analysis. The quantile–quantile (QQ) plot was also drawn, which showed no evidence of systematically inflation of the G + T test (Fig. 1).

**Table 1:** Summary of top 5 genes associated with SBP, DBP, and HTN

	Chr	BP1	BP2	Gene	P_G+T_	P_G_	P_T_	#SNV
SBP	1	27763371	27777626	TMEM222	1.94E-003	**1.61E-004**	5.87E-001	100
SBP	17	5486374	5490814	MIS12	**1.63E-004**	2.26E-003	4.46E-003	134
SBP	19	10528205	10581112	PDE4A	3.99E-004	1.30E-001	**2.39E-004**	311
SBP	3	57376936	57627630	DNAH12	7.87E-003	**2.65E-004**	9.92E-001	1691
SBP	19	32291486	32313186	MAG	1.74E-003	7.61E-001	**3.22E-004**	216
DBP	11	72239077	72244176	PHOX2A	1.45E-005	4.17E-001	**5.22E-006**	112
DBP	19	58182989	58213562	ZNF274	3.68E-004	4.03E-001	**1.27E-004**	234
DBP	3	10277571	10284767	GHRL	1.52E-004	4.94E-002	**1.46E-004**	118
DBP	11	43991253	44040694	ALX4	**4.16E-004**	1.16E-003	2.20E-002	458
DBP	1	61681046	61725423	TM2D1	5.52E-003	**5.43E-004**	7.86E-001	307
HTN	3	183042973	183066541	TRA2B	**1.06E-005**	4.91E-003	3.11E-005	496
HTN	7	27445802	27583281	HIBADH	4.05E-004	**3.31E-005**	7.38E-001	204
HTN	17	4004445	4143020	ZZEF1	**3.86E-005**	2.24E-002	1.27E-004	871
HTN	3	57277865	57480169	DNAH12	5.23E-003	**1.13E-004**	9.99E-001	327
HTN	5	131138142	131164051	SLC22A5	2.58E-003	**1.34E-004**	9.12E-001	151

*BP1* first base pair of the gene, *BP2* last base pair of the gene, *Chr* chromosome, *P*_*G*_ *, P*_*G+T*_ *, P*_*T*_ *p* values from 3 types of tests, and the bolded *p* value is the smallest of the 3 p values, *#SNV* number of single nucleotide variants in the gene region

## Discussion

Further investigation of the top genes also found biological plausibility of several genes related to blood pressure. For instance, the product of *PED4A* hydrolyzes the second messenger cyclic adenosine monophosphate (cAMP), which plays a crucial role in controlling blood pressure [11]. *PHOX2A* is also important for the development of autonomic nervous system, which controls the involuntary functions, such as heart rate and blood pressure [12].

The study has certain limitations. Out of 8,348,674 SNVs, 1,391,764 (17 %) were unused because they are not in or near any gene. We could group these SNVs by physical location and also incorporate them into the analysis [4]. We found limited association evidence of single-nucleotide polymorphisms (SNPs) identified from previous GWAS, possibly because of differences in study samples (ie, whites vs. Mexican Americans). Another possibility is that majority of SNVs in our study are rare (MAF <0.01), whereas previous GWAS mainly focus on common variants (MAF >0.05).

The analysis of a large number of genes raised the issue of multiple testing. In our analysis, the false discovery rate approach was used to account for the issue of multiple testing. After adjusting for multiple testing, none of the genes could reach statistical significance. By using the biology knowledge and statistical tools, we might be able to further reduce the number of tests and increase our chance to detect an association. For instance, all assembles of one gene have high correlation, and we can either exclusively use the primary assemble or adjust *p* values for multiple correlated tests to better solve the multiple testing issue.

## Conclusions

The emerging sequencing data and other omic data provide invaluable source for genetic study of human diseases, yet integrating and modeling these high-dimensional data remain a great challenge. By integrating both sequencing variants and gene expression into the association analysis, the weighted U approach provides a powerful and computationally efficient way for screening disease-associated genes. By applying the approach to the GAW19 data, we showed that the joint analysis of sequencing variants and gene expression could have some advantages over association analysis only using sequence variants or gene expression.
